# Using iterative learning to improve understanding during the informed consent process in a South African psychiatric genomics study

**DOI:** 10.1371/journal.pone.0188466

**Published:** 2017-11-29

**Authors:** Megan M. Campbell, Ezra Susser, Sumaya Mall, Sibonile G. Mqulwana, Michael M. Mndini, Odwa A. Ntola, Mohamed Nagdee, Zukiswa Zingela, Stephanus Van Wyk, Dan J. Stein

**Affiliations:** 1 Department of Psychiatry and Mental Health, University of Cape Town, Cape Town, South Africa; 2 Department of Epidemiology. Joseph Mailman School of Public Health, Columbia University and New York State Psychiatric Institute, New York; 3 Department of Psychology, Rhodes University and Fort England Hospital, Grahamstown, South Africa; 4 Department of Psychiatry and Behavioural Sciences, Walter Sisulu University, Mthatha, South Africa; 5 Department of Psychiatry and Mental Health, University of Cape Town, South Africa, and the MRC Unit on Risk and Resilience in Mental Disorders; University of Liverpool, UNITED KINGDOM

## Abstract

Obtaining informed consent is a great challenge in global health research. There is a need for tools that can screen for and improve potential research participants’ understanding of the research study at the time of recruitment. Limited empirical research has been conducted in low and middle income countries, evaluating informed consent processes in genomics research. We sought to investigate the quality of informed consent obtained in a South African psychiatric genomics study. A Xhosa language version of the University of California, San Diego Brief Assessment of Capacity to Consent Questionnaire (UBACC) was used to screen for capacity to consent and improve understanding through iterative learning in a sample of 528 Xhosa people with schizophrenia and 528 controls. We address two questions: firstly, whether research participants’ understanding of the research study improved through iterative learning; and secondly, what were predictors for better understanding of the research study at the initial screening? During screening 290 (55%) cases and 172 (33%) controls scored below the 14.5 cut-off for acceptable understanding of the research study elements, however after iterative learning only 38 (7%) cases and 13 (2.5%) controls continued to score below this cut-off. Significant variables associated with increased understanding of the consent included the psychiatric nurse recruiter conducting the consent screening, higher participant level of education, and being a control. The UBACC proved an effective tool to improve understanding of research study elements during consent, for both cases and controls. The tool holds utility for complex studies such as those involving genomics, where iterative learning can be used to make significant improvements in understanding of research study elements. The UBACC may be particularly important in groups with severe mental illness and lower education levels. Study recruiters play a significant role in managing the quality of the informed consent process.

## Introduction

Obtaining informed consent is a great challenge in global health research. The understanding of research study elements, such as the purpose of the research, risks and benefits of participation and the research design, varies considerably amongst participants in both high-income countries (HICs) and low-middle income countries (LMICs) [1]. A recent meta-analysis of informed consent comprehension in health research conducted in Sub-Saharan Africa indicates that only 65% of participants generally understand the purpose of a research study, and only 51% appreciate the risks of participation [2]. There is a need for tools that can screen for and improve potential research participants’ understanding of the research study at the time of recruitment. However, limited empirical research has been conducted in LMICs on evaluating informed consent processes. Available studies indicate that low literacy levels and lengthy informed consent materials complicate the informed consent process, particularly when drawing from complex medical terminology that is incongruent with local cultural idioms and expressions [2, 3]. Many participants also tend to be unfamiliar with the research process, and power dynamics between recruiting clinicians and potential research participants may influence the voluntariness of participation [1, 2, 4]. In the context of these challenges we sought to investigate the quality of informed consent obtained during the course of a South African psychiatric genomics study, by examining whether participants’ understanding was improved by iterative learning during recruitment, as well as factors that influenced participants’ understanding.

Studies in HICs show that understanding of informed consent materials can be improved through an iterative learning process of firstly presenting the study information, secondly assessing a participant’s understanding of the study elements, and finally revisiting and revising poorly understood elements [5–7]. Participant understanding has been shown to be reliably assessed using the University of California, San Diego Brief Assessment of Capacity to Consent Questionnaire (UBACC), which was originally developed as a 10-item screening tool for decisional capacity in participants being recruited for schizophrenia research [8]. The tool may, however, have broader applicability in global health research. The UBACC screens for participants’ appreciation and understanding of key research study elements including the purpose of the study, protocol procedures, study risks and benefits, and the voluntary nature of participation [8]. In addition to schizophrenia research [9] the UBACC has been used as a screening tool for decisional capacity in neurocognitive [10–12] and HIV [13] research. To the authors’ knowledge, however, the UBACC has yet to be used in LMICs.

We used the UBACC as a screening and education tool during recruitment of cases and controls for the Genomics of Schizophrenia in South African Xhosa People (SAX) project. We address two questions: firstly, whether research participants’ understanding of the research study improved through iterative learning conducted after screening at the time of recruitment; and secondly, what were predictors for better understanding of the research study at the initial screening?

## Methods

### Genomics of schizophrenia in South African Xhosa People (SAX)

African populations are currently under-represented in psychiatric genomics research [14]. The SAX project aims to contribute to filling this gap by identifying mutations underlying predisposition to schizophrenia in the Xhosa people. Schizophrenia is characterized by a range of cognitive, behavioral and emotional dysfunctions and by impaired occupational and social functioning [15]. These impairments could have implications for decisional capacity in consenting to participate in research. Studies in HICs indicate that decisional capacity varies considerably across people with schizophrenia, influenced most significantly by the neurocognitive impairments associated with the disorder [16–18]. The SAX study provided a unique opportunity to study the consent process in a LMIC, both among people with severe mental illness (cases) and among people presenting for treatment for other health-related problems (controls).

SAX cases are recruited from provincial psychiatric hospitals and clinics in the Eastern and Western Cape provinces of South Africa, and include those who have fulfilled the diagnostic criteria for schizophrenia or schizoaffective disorder over at least a two-year period. SAX controls include Xhosa people who present for treatment of other medical conditions at university-affiliated general medical hospitals and community health centers that draw from similar catchment areas to the psychiatric hospitals. Exclusion criteria for controls include a previous history of psychosis, pre-existing neurological conditions such as brain injury or epilepsy, or patients presenting for treatment of injuries related to substance use.

All participants recruited for the SAX study complete the Structured Diagnostic Interview for DSM-IV Axis I Disorders (SCID-I) [19], along with other psychiatric rating scales, and provide blood samples for DNA and HIV testing. All clinical interview materials are administered in Xhosa, having been translated in accordance with the World Health Organization (WHO) translation guidelines [20]. This process includes both forward and back-translation of the tools, as well as a committee approach of discussion and debate about challenging concepts and constructs to select the most linguistically and conceptually appropriate Xhosa terminology. However, in some instances no direct Xhosa translation is available, particularly for the more complex genomics terminology, and translations then focused on developing phrases or Xhosa terminology that captured the conceptual explanation of these English terms. Essentially translators are developing a discourse for neurogenetic concepts in the Xhosa language.

Participants complete the UBACC during the informed consent process. This procedure, described under data collection, assists us in identifying SAX study elements that require further explanation. Participants then engage in an iterative learning process of corrective feedback and repetition of information to improve understanding of these elements.

This research was approved by the University of Cape Town, Health Sciences Research Ethics Committee, Rhodes University Ethics Standards Committee, Walter Sisulu University, Faculty of Health Sciences Research Ethics Committee, Columbia University Institutional Review Board and University of Washington Human Subjects Division.

### Hypotheses

While studies have established that people with schizophrenia will likely struggle more with informed consent understanding than healthy controls [16–18], work within LMICs highlights the influence of poor literacy and unfamiliarity with the research process as additional factors that impact on the informed consent process [2–4]. The lack of local language terms to further support the explanation of genomics terminology is an additional barrier. We therefore anticipated that both cases and controls would struggle with some of the terminology and concepts in the SAX protocol such as genetics, DNA, sample storage and cell immortalization. This may compromise understanding of key SAX study elements such as the purpose of the research, risks and benefits of participation and the research design. We hypothesized that an iterative learning approach, using the UBACC would improve understanding among both cases and controls.

Second, previous studies identified schizophrenia diagnosis and neuro-cognitive impairments as predictors of poor decisional capacity [16, 17]. We hypothesized that being a case or control, levels of education and global functioning scores on the Global Assessment of Functioning (GAF) scale [21], would be associated with understanding of SAX study elements during initial screening with the UBACC. We were also interested in associations between the SAX recruiters, the region within which participants were recruited from as well as sex and age demographics, and understanding of the SAX study element.

### Data collection

During recruitment, all SAX participants receive information about the SAX study aims, methods, risks and benefits of participating, and expected outcomes in the form of an information sheet written in Xhosa. This material is read and explained to each participant by one of five first language Xhosa speaking SAX psychiatric nurse recruiters, with special focus on complex terms such as genetics, DNA, sample storage and cell immortalization. For the purposes of this study we then ask participants to complete the UBACC as a screening of their initial understanding of the study. Individual UBACC items that are incorrectly answered are flagged for follow-up with the participant. The recruiter then immediately revisits the SAX study elements related to those items, corrects the participant’s misunderstanding and repeats important information about that study element, often by rephrasing the material. The incorrect UBACC items are re-administered during a second UBACC administration. Participants achieving a total score of above 14.5 demonstrate sufficient understanding of the study elements to indicate capacity to consent [8]. Participants who continue to score below this cut-off after the second UBACC administration receive additional corrective feedback and information until the cut-off score is achieved, or a total of 4 administrations of the UBACC are completed. This process takes between 15–30 minutes. Participants who continue to score below the 14.5 cut-off are then removed from the study.

Participants recruited for the SAX study from March 2015 to November 2016 were included for analysis. Cases and controls were matched to sex, age, education level and region recruited from. To achieve a power of 0.9 at a significance of 0.05 we required a sample size of at least 181 matched pairs. The initial UBACC screening scores and scores obtained after an iterative learning process were recorded, along with the Global Assessment of Functioning (GAF) score and the following participant information: diagnosis, gender, age group, education level, region recruited from, and the psychiatric nurse doing the recruiting.

### Measures

#### The University of California, San Diego Brief Assessment of Capacity to Consent Questionnaire (UBACC)

The 10-item UBACC questionnaire was translated into Xhosa and then adapted for the SAX study, including one change. Item 10 of the original UBACC (“Who will pay for your medical care if you are injured as a direct result of participating in this study?”) was not applicable because the SAX study was an exploratory study that provided little risk of injury to participants. This item was replaced with a question about cell line immortalization which was a key aspect of the SAX study (“Do you have to agree to store your cells?”). A summary of the original UBACC English version, the translated and adapted SAX Xhosa version and English back translation of the tool are summarized in Table 1. Each item is scored on a scale of 0 (incorrect), 1 (some understanding) or 2 (full understanding); with a maximum score of 2 for each item(total scores therefore ranging from 0 to 20).

**Table 1:** The forward- and back-translation of the UBACC.

Original UBACC English version	UBACC Xhosa version	UBACC English back-translation
1. What is the purpose of the study that was just described to you?	1. Yintoni injongo yoluphondo lwenzziwayo ndigqiba ukukucacisela ngalo?	1. What is the purpose of the investigation that I have just explained to you?
2. What makes you want to consider participating in this study?	2. Yintoni ekwenze ukuba ufune ukuthabatha inxaxheba koluphando?	2. What made you want to take part in this investigation?
3. Do you believe this is primarily research or primarily treatment?	3. Ingaba ucinga ukuba olu luphando okanye lunyango?	3. Do you think this is an investigation or treatment?
4. Do you have to be in this study if you do not want to participate?	4. Ucinga ukuba kunyanzelekile na ukuba ube koluphando nokuba awufuni?	4. Do you think you must take part in this investigation even if you don’t want to?
5. If you withdraw from the study, will you still be able to receive regular treatment?	5. Ucinga ukuba uye wayeka ukuthabatha inxaxheba koluphando ungakwazi ukufumana unyango lwakho njengesiqhelo?	5. Do you think if you stop participating in this investigation you won’t be able to get your treatment as usual?
6. If you participate in this study, what are some of the things that you will be asked to do?	6. Ukuba uthe wathatha inxaxheba koluphando zeziphi ezinye zezinto ozakucelwa uzenze?	6. If you are taking part in this investigation what are the things that you will be asked to do?
7. Please describe some of the risks or discomforts that people may experience if they participate in this study	7. Ndicela uchaze ubungozi okanye ubunzima onokubufumana ukuba uthe wathatha inxaxheba koluphando?	7. Can you please explain some of the dangers, risks, harms or difficulties you may come across if you take part in this investigation?
8. Please describe some of the possible benefits of this study	8. Ndicela uchaze inzuzo/amanye amancedo anokufumaneka koluphando?	8. Can you explain the benefits, advice, help or other services you will get from this investigation?
9. Is it possible that being in this study will not have any benefit to you?	9. Ingaba igenzeka into yokuba oluphando lungangabi luncedo kuwe?	9. Do you think that the investigation may not help you?
10. Who will pay for your medical care if you are injured as a direct result of participating in this study? Replaced with: Do you have to agree to store your cells?	10. Ingaba kunyanzelekile ukuba imisebe yakho yegazi iyokugcinwa?	10. Do you have to agree to store your cells?

#### Global Assessment of Functioning (GAF) scale

The GAF scale is a standardized method of assessing the severity of psychiatric illness against overall level of functioning (on a scale of 0–100) [21]. While this measure is typically used in psychiatric populations, we included GAF scores for our controls as mental and substance use disorders are commonly reported amongst people presenting with health complaints.

#### Additional participant information

The following categorical variables were assigned to participants: diagnosis (cases–diagnosis of schizophrenia or schizoaffective disorder; or control), gender (male or female), age group (20–39 years or 40–59 years), education level (primary school–achieved Grade 7 or less; secondary school–achieved Grade 8 or more), region recruited from (Eastern Cape or Western Cape). Dummy codes were developed for each psychiatric nurse recruiter (A-E). The recruiter (A) with the lowest UBACC screening scores was assigned as the reference nurse against which the other four nurses were compared.

### Data analysis

A repeated measures t-test was used to compare performance on the UBACC across trials 1 (screening) and 2 (after iterative learning) in our cases and controls respectively. Linear regression methods were used to identify hypothesized predictors of decisional capacity during screening with the UBACC. The following variables were included in the model for analysis: UBACC scores from trial 1; GAF scores; participant being a case versus a control; sex; age group; education level; region recruited from; and psychiatric nurse conducting the recruitment interview. Statistical significance levels for both analyses were set at 5%, and data analysis was generated and managed using SPSS.

## Results

A total of 1056 participants, or 528 matched pairs of cases and controls were included for analysis. Sample information is summarized in Table 2.

**Table 2:** Sample information.

Variables	Categories/Labels	Frequencies (%)
**Diagnosis:**	Case	528 (50%)
	Control	528 (50%)
**Sex:**	Male	946 (89.6%)
	Female	110 (10.4%)
**Age group:**	Younger (20–39)	674 (63.8%)
	Older (40–59)	382 (36.2%)
**Education level:**	Primary (≤Grade 7)	336 (31.8%)
	Secondary (≥Grade 8)	720 (68.2%)
**Region recruited from:**	Western Cape	276 (26.1%)
	Eastern Cape	780 (73.9%)
**Psychiatric nurse recruiter**	A	311 (29.5%)
	B	285 (27.0%)
	C	198 (18.8%)
	D	116 (11.0%)
	E	146 (13.8%)

**Fig 1:**
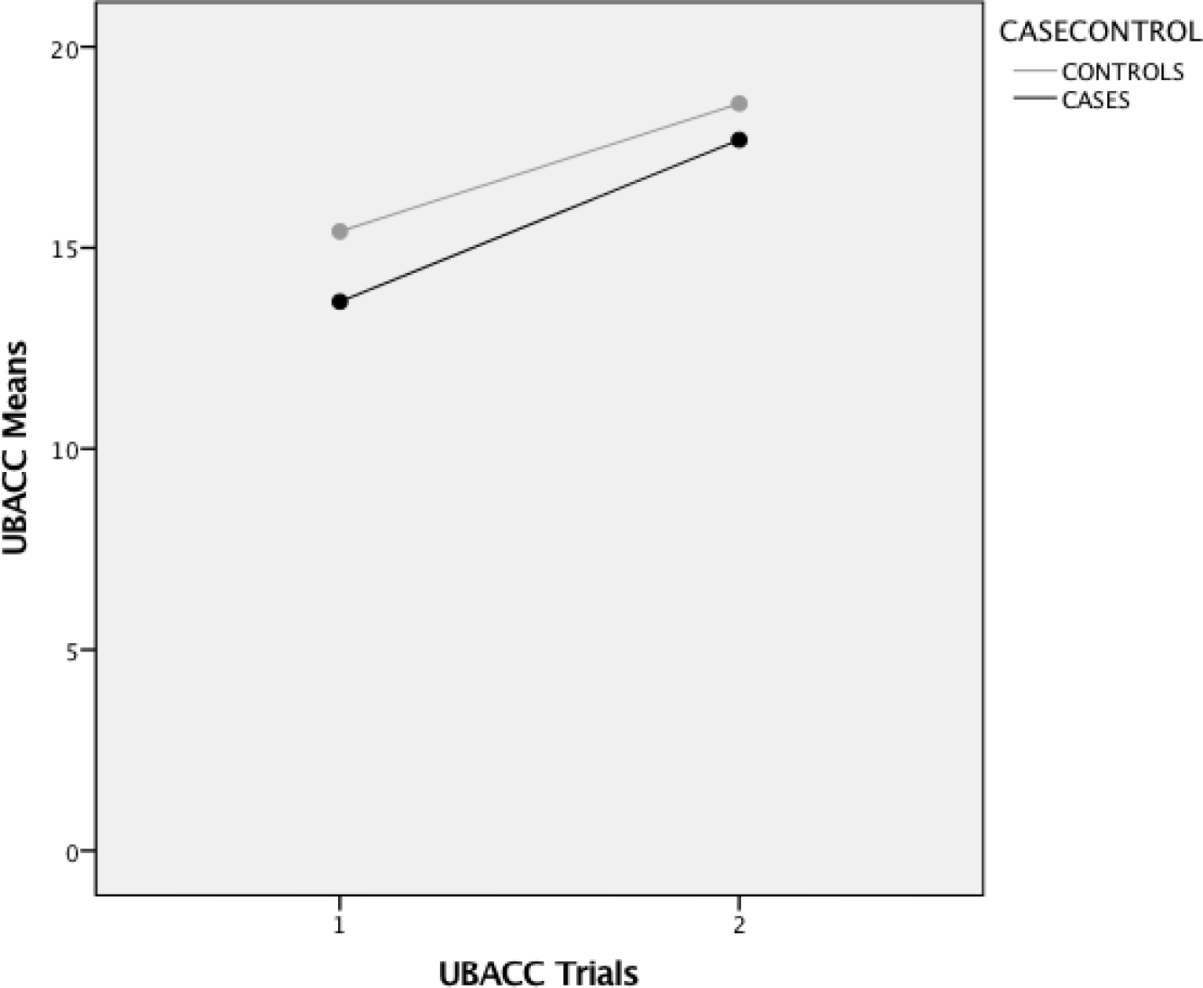
Changes in UBACC means across trials 1 and 2.

Results from the repeated measures t-test, are presented in Table 3 and Fig 1. Both cases and controls achieved large and significant improvements in UBACC scores following iterative learning. Importantly, 290 (55%) cases and 172 (33%) controls scored below the 14.5 cut-off for acceptable understanding of the research study elements during screening, and without iterative learning would have been excluded from the study. After iterative learning, however, only 38 (7%) cases and 13 (2.5%) continued to score below this cut-off after the second UBACC trial. These participants engaged in further iterative learning and up to two additional UBACC trials, following which all achieved above the 14.5 cut-off score and were recruited for the study.

**Table 3:** Comparison of UBACC mean scores for cases and controls across UBACC trials 1 and 2.

	Trial 1 M(SD)	Trial 2 M(SD)	Mean difference	Correlation	P-value	Effect size
**Cases**	13.66 (3.5)	17.69 (2.1)	4.03 (2.5)	0.700	<0.000	1.240
**Controls**	15.41 (3.0)	18.59 (1.7)	3.18 (2.2)	0.695	<0.000	1.128

Results from the linear regression, summarized in Table 4, demonstrated large significant associations between understanding of SAX study elements during screening, and i) participants without a diagnosis of schizophrenia, ii) those with a higher level of education, and iii) specific psychiatric nurse recruiters explaining the study. Being female, and of younger age predicted better understanding as did higher GAF scores, whilst the region participants were recruited from was not significant.

**Table 4:** Linear regression analysis of hypothesized predictors of decisional capacity using the UBACC.

Model		Unstandardized co-efficient Beta	Std. Error	P-value	CI:95%
**Constant**		10.345	0.494	<0.000	9.376–11.314
Diagnosis (Cases*/Controls)		1.541	0.199	<0.000	1.151–1.931
**GAF score**		0.027	0.007	<0.000	0.014–0.040
Sex (Male*/Female)		0.643	0.293	0.028	0.069–1.218
Age (Younger/Older*)		0.710	0.189	<0.000	0.338–1.082
Education (Primary*/Secondary)		1.372	0.194	<0.000	0.992–1.752
Region (WC*/EC)		0.186	0.206	0.368	-0.219–0.590
Psychiatric nurse recruiter (A*)	B	2.403	0.235	<0.000	1.942–2.864
	C	2.426	0.262	<0.000	1.911–2.941
	D	3.405	0.327	<0.000	2.762–4.047
	E	1.628	0.301	<0.000	1.038–2.218

* Reference Group

## Discussion

Three key points emerge from these results. First, congruent with existing literature from HICs [5, 17] our findings support the growing evidence demonstrating the ability to improve decisional capacity through an iterative learning process using the UBACC. The UBACC proved an effective tool for improving understanding of SAX research study elements during recruitment of both cases and controls in our LMIC setting. This appeared to be the case despite the inherent challenges with low literacy, poor education and complex research study terminology that were often not supported in the local language [2, 3]. The UBACC assisted study recruiters in determining how much of the SAX protocol was understood by participants after an initial introduction to the material, and who would particularly benefit from iterative learning. It also allowed recruiters to identify challenging elements of the SAX study, and through iterative learning, improve understanding of these elements. This informed consent process holds valuable utility for other psychiatric genomics studies in LMICs and African settings. However, improvements in quality of understanding during consent may have wider implications, one being the influence on recruitment rates. Improved understanding of the field of genomics and how participating in such research may benefit society in the longer term may result in an increased willingness on behalf of individuals to enroll on a study. Although greater understanding of the study risks or limited direct benefits to communities in the shorter term may have the opposite outcome with more individuals choosing not to participate in a study.

Second, improvements in quality of understanding during consent in our control group suggest that the UBACC and iterative learning may be helpful in other genomics studies and the UBACC hassubsequently been incorporated as a recommended tool for evaluating quality of consent in the Human Heredity and Health Consortium (H3Africa) Revised Guidelines for Informed Consent [22]. People with severe mental illness are typically classified as a vulnerable group for research. Yet many LMICs contend with poverty, challenges in access to education and healthcare that create their own layers of vulnerability. There is a need for standardized informed consent screening tools that can be used on site, during recruitment to improve quality of consent. Such tools need to be easily adaptable to the diverse cultural and language needs of LMIC research settings [2]. The UBACC appears particularly useful in this regard because questionnaire items are adapted to the specific research study underway, allowing for cultural and language translation to be performed concurrently.

Third a diagnosis of schizophrenia and low education level were strong predictors of poorer understandings of SAX study elements initially, and to a lesser extent lower GAF scores and older age. Such participants are likely to require more time and resources during recruitment. However, the individual characteristics of study recruiters proved to be the largest predictor of participants’ understanding of SAX study elements, a finding that is consistent with other studies in LMICs [2, 4]. The understanding the recruiter holds about the study elements, their ability to improvise and simplify explanations and the clarity with which they are able to explain this information to participants, often moving between English and the local language in doing so, likely has an important impact on the quality of informed consent. Adequate and continued training of recruitment staff about the research study elements, informed consent procedures and consistency in evaluating these elements using the UBACC is clearly of value.

### Limitations

Several limitations of the current analysis and outcomes should be highlighted. First, our sample, while representative of the larger SAX sample, comprised mostly male participants (90%) and did not adequately represent Xhosa women with schizophrenia. One explanation for this may be that our recruitment strategy focused on participants from state psychiatric hospitals and clinics where majority of the patients with a diagnosis of schizophrenia were men. Second, the translation of psychometric instruments across different languages and cultural contexts, and achieving linguistic and conceptual equivalence across English and Xhosa language versions of psychiatric tools holds many challenges [23, 24]. The lack of specific Xhosa terminology for genomics language means that translators are developing this discourse as studies are implemented in African settings. This may result is confusion and misunderstanding, which will impact on the quality of understanding and informed consent. Regular revising of informed consent materials and specifically the information sheet distributed to recruited participants is an important contingency measure. Third, the specific characteristics, temperament, and communicative skills of individual recruiters are likely to have impacted on UBACC scores e.g. some may have been more meticulous, knowledgable and provided explanations about the SAX study materials in more accessible ways than others (though such variables are difficult to quantify accurately). Finally, with respect to predictors of understanding during screening, confounding variables such as neuro-cognitive impairment were not taken into account during analysis.

## Conclusion

Our analysis indicates that the UBACC is an effective tool for improving understanding of research study elements during recruitment in a LMIC setting. Improved understanding was evident both among cases and controls. The UBACC holds valuable potential utility in global health research, and particularly for improving understanding during informed consent processes in LMIC health research. This is particularly so for participants with severe mental illness, and those with low education levels. Finally, the study recruiters play a significant role in managing the quality of the informed consent process.

## Supporting information

S1 Text Supporting data. (XLSX)
